# Energy Landscapes and Structural Ensembles of Glucagon-like Peptide-1 Monomers

**DOI:** 10.1021/acs.jpcb.4c01794

**Published:** 2024-06-04

**Authors:** Alasdair D. Keith, Eva Přáda Brichtová, Jack G. Barber, David J. Wales, Sophie E. Jackson, Konstantin Röder

**Affiliations:** † Yusuf Hamied Department of Chemistry, University of Cambridge, Lensfield Road, Cambridge CB2 1EW, U.K.; ‡ Now: Department of Biochemistry, School of Medicine, Emory University, 1510 Clifton Rd NE, Atlanta, Georgia 30322, United States; § Now: Institute of Chemical, Environmental and Bioscience Engineering, Technische Universität Wien, Gumpendorferstr. 1A, Vienna 1060, Austria; ∥ Now: Randall Centre for Cell & Molecular Biophysics, King’s College London, Great Maze Pond, London SE1 1UL, U.K.

## Abstract

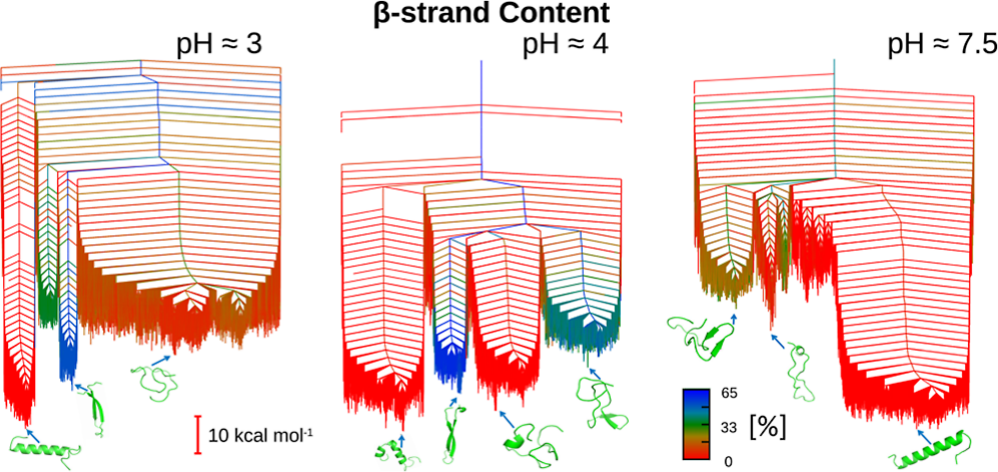

While GLP-1 and its analogues are important pharmaceutical agents in the treatment of type 2 diabetes and obesity, their susceptibility to aggregate into amyloid fibrils poses a significant safety issue. Many factors may contribute to the aggregation propensity, including pH. While it is known that the monomeric structure of GLP-1 has a strong impact on primary nucleation, probing its diverse structural ensemble is challenging. Here, we investigated the monomer structural ensembles at pH 3, 4, and 7.5 using state-of-the-art computational methods in combination with experimental data. We found significant stabilization of β-strand structures and destabilization of helical structures at lower pH, correlating with observed aggregation lag times, which are lower under these conditions. We further identified helical defects at pH 4, which led to the fastest observed aggregation, in agreement with our far-UV circular dichroism data. The detailed atomistic structures that result from the computational studies help to rationalize the experimental results on the aggregation propensity of GLP-1. This work provides a new insight into the pH-dependence of monomeric structural ensembles of GLP-1 and connects them to experimental observations.

## Introduction

Glucagon-like peptide-1 (GLP-1) is a peptide hormone comprising 30 or 31 amino acids.^1−3^ As an incretin, GLP-1 stimulates a decrease in blood glucose levels and, thus, is of increasing importance within the pharmaceutical industry as a therapeutic agent for the treatment of type 2 diabetes and obesity.^4−6^ Several GLP-1 analogues have been produced commercially.^7,8^ Though currently in widespread use as an intravenous treatment, challenges remain with regard to its formulation. Recent studies have demonstrated that a selection of such GLP-1 receptor agonists can be administered orally,^9−12^ a potential breakthrough in diabetic treatment, which could circumvent the requirement for intravenous insulin therapy. However, care must be taken in applying GLP-1-based treatments due to the susceptibility of these peptides to self-associate into amyloid fibrils over a broad range of conditions.^3,13,14^

Amyloid fibril formation is a complex, multistep process. Classical fibrillation of peptides exhibits three distinct phases within an overall sigmoidal kinetic profile: a lag phase, in which primary nucleation occurs among other processes; a growth phase, which encompasses elongation and secondary nucleation processes; and a plateau phase, in which the monomer is depleted, or equilibrium is achieved.^15−18^ In the lag phase, the monomer concentration is high, whereas the concentration of fibrils rapidly increases throughout the growth phase. In the plateau phase, the fibril species dominate.

Of crucial importance to fibrillation is the formation of oligomeric species from the original monomers. In general, such oligomers exhibit a wide variety of morphologies, resulting in a broad range of half-lives and stabilities.^19−23^ These oligomers can essentially be split into two classes, namely “on-pathway” oligomers, which are capable of being elongated into amyloid fibrils, or “off-pathway” oligomers, which arise from side-reactions and cannot be directly elongated to give amyloid fibrils. Although some long-lived oligomers have been identified,^22,24−26^ most species tend to be transient in nature, making their detection and structural characterization challenging.^18,27,28^ Therefore, to better understand this complex fibrillation process, a deeper knowledge of the original monomer structures, which can be more easily isolated, is crucial. These monomers exhibit a range of structures whose relative stabilities vary with respect to the precise physiological conditions, so an understanding of the structural response to these conditions can be used to infer the relative propensity of aggregation. In other words, condition-dependent features of amyloid fibril formation can be inferred from the monomer stage.

Furthermore, in contrast to the classical description of a nucleation-propagation mechanism, where increasing the peptide concentration decreases the lag time and half-life for aggregation, increasing the concentration of the GLP-1 monomer under specific conditions (specifically, at pH 7.5) leads to an increased lag time and half-life.^3^ This unusual feature of peptide dependence, which was determined by Zapadka et al. using thioflavin T fluorescence assays,^3^ has been attributed to an off-pathway self-assembly process, which is likely to depend on the starting monomer conformation. Their data revealed that there is a strong pH-dependence to the unusual kinetic behavior observed, and it was suggested from p*K*_a_ studies that this property could be due to protonation/deprotonation of the N-terminus. Later studies on GLP-1 at other pH values and on a C-terminally amidated form of GLP-1 showed that net charge is likely a factor in determining whether off-pathway species are formed.^29^ In addition, more recent studies have shown that the stable, off-pathway oligomer formed under some conditions can inhibit aggregation.^26^ What is lacking in these studies is an understanding of how pH changes the structural ensemble of GLP-1, thus facilitating the population of either on- or off-pathway oligomers. This problem is due to the inherent difficulty in obtaining detailed structural information on ensembles of partially structured and interconverting monomeric species populated at different pH values via conventional biophysical methods.^3^

Indeed, analyzing the structural ensemble of monomer solutions presents a complicated biophysical challenge. Spectroscopic data are typically difficult to deconvolute given the number of distinct yet related structural motifs open to these peptides, which is further complicated by their dynamic, condition-dependent variation within a solution.

A complementary way to study the structural ensembles adopted by the monomers is the use of molecular simulations, which allow for the resolution of the different structural motifs adopted. However, many simulation methods are also hampered by broken ergodicity and cannot access the long time scales associated with biomolecular motion.^30^ The computational energy landscape framework^31,32^ circumvents this issue and enables exploration of the full underlying molecular energy landscape. Structural, thermodynamic, and kinetic data can be readily obtained.^33^ In the past, this simulation framework has been used successfully to study amyloidogenic peptides,^34−36^ and provides insights into the nature of the disorder exhibited by peptides.^37^

In the present work, we explore the energy landscapes of GLP-1 across a range of pH values to determine the relative stabilities of the different structures. GLP-1 structure can respond significantly to even small variations from the physiological pH of 7.4,^3^ so it is important to understand how the GLP-1 monomer responds to such pH variation. The 7–36 and 7–37 forms of GLP-1 (the latter contains an extra glycine at the C-terminus) are considered here. We connect the simulation data to structural data from far-UV CD spectroscopy and aggregation propensities from thioflavin T assays conducted at pH 3, 4, and 7.5. Our results are consistent with the biophysical data of Zapadka et al.,^3^ and reveal interesting new details with regard to relative conformational stabilities, which should assist and help to clarify future experimental studies on GLP-1 monomer structure and its link to aggregation propensity.

## Methods

### Exploring the Energy Landscape

Discrete path sampling^38,39^ was employed to create kinetic transition networks^40,41^ comprising local minima and the transition states that connect them. The doubly-nudged elastic band (DNEB) algorithm^42−45^ was used to generate transition state candidates and hybrid eigenvector-following (HEF)^46^ to converge them. Approximate steepest-descent paths were used to obtain the minima directly connected to these transition states. A customized L-BFGS approach^47,48^ with an RMS force convergence criterion of 10^–6^ kcal mol^–1^ was employed. The databases were then refined by finding alternative pathways (SHORTCUT^49,50^ scheme), removing kinetic traps (UNTRAP^50^ scheme), and local sampling (CONNECTUNC^36^ scheme). Disconnectivity graphs^51−54^ are used for graphical representation of potential and free energy landscapes. Further details can be found in various reviews.^31,55,56^ Secondary structure content from DSSP and the radius of gyration are calculated with Cpptraj.^57^

### Starting Points and Force Field for Simulations

The energy landscapes were explored for seven distinct conditions, which are detailed in Tables 1 and S1. All calculations employed the AMBER ff14SB^58^ force field, properly symmetrized, as in previous studies.^36,59,60^ An implicit generalized Born solvent model (igb = 8)^61^ was used with the Debye–Hückel approximation for salt (0.1 M).

**Table 1:**
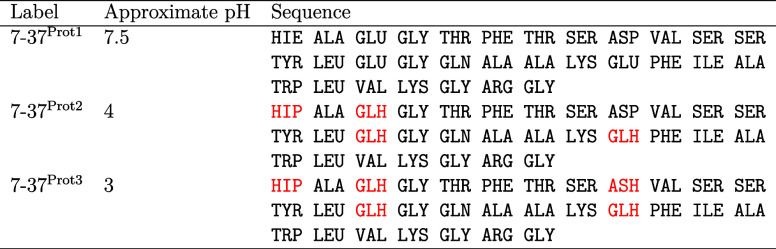
Sequence and Protonation States of GLP-1 Variants Studied^a^

a Approximate pH values are given so that simulations can be more readily compared to experiment. Three-letter labels for residues indicating the state of protonation/deprotonation follow the convention developed by the AMBER package. Here, histidine is represented not by HIS but by HIE if the residue is deprotonated and has hydrogen on the epsilon nitrogen only and by HIP if the residue is protonated and so has hydrogen atoms on both the delta and epsilon nitrogens. GLU represents glutamic acid in its deprotonated form, whereas GLH represents this residue when protonated. Similarly, ASP represents deprotonated aspartic acid, whereas ASH represents this residue in its protonated form. Residues highlighted in red are those that differ from 7–37^Prot1^.

**Figure 1:**
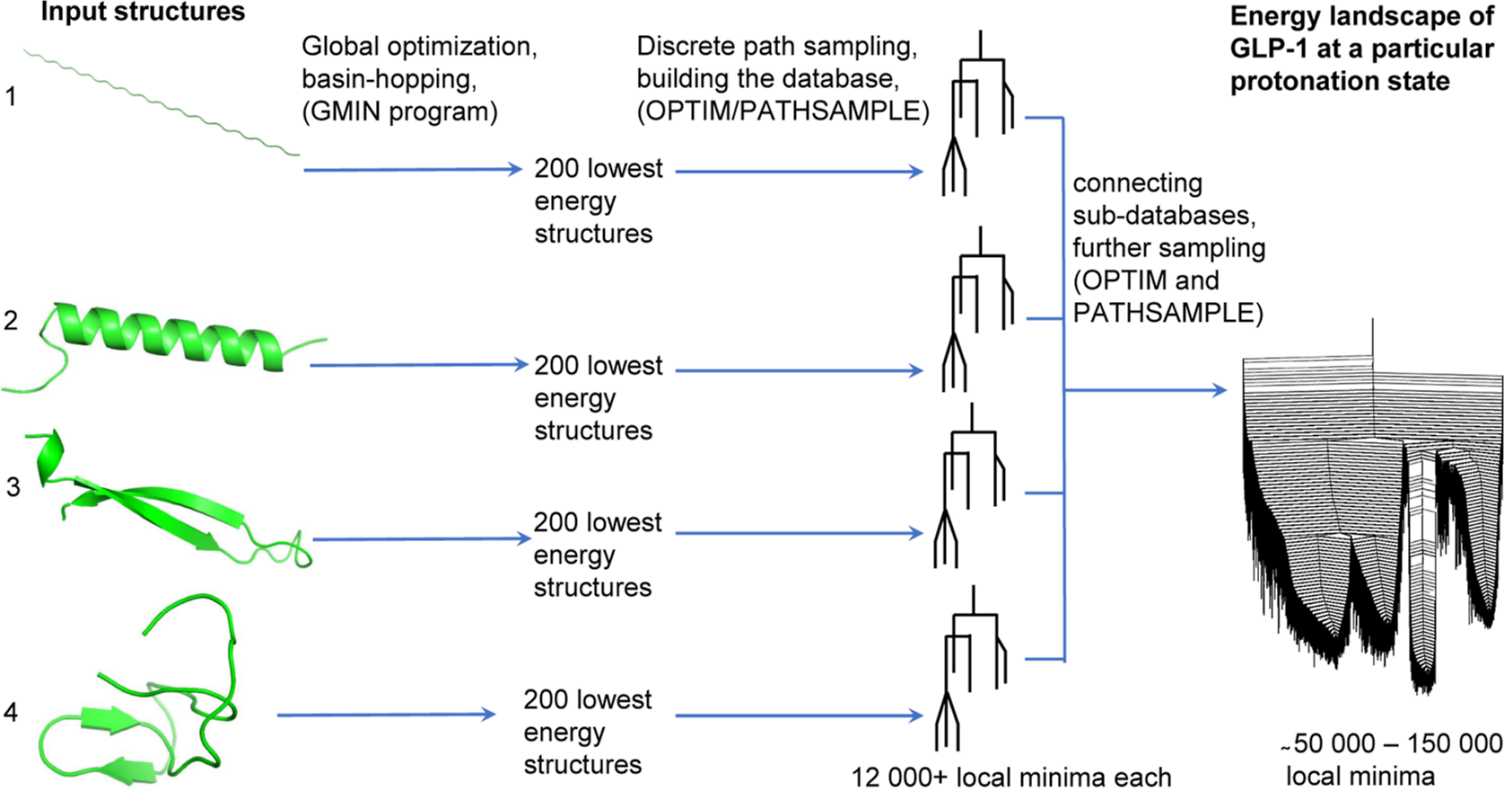
Setup for the energy landscape explorations: four distinct structures were used to seed basin-hopping global optimization. After the searches, the 200 lowest structures of each were used to initialize discrete path sampling, and the final databases were obtained by combining these subdatabases.

For the GLP-1 (7–37) structures, two starting structures were generated from sequence using LEAP. Basin-hopping^62−64^ global optimization was used on both of them to obtain low energy structures (60,000 steps with rotamer moves^a^). The two lowest structures obtained were two helices connected by a β-turn and two β-strands connected by a β-turn. Low energy structures from this system were then used as seeds for basin-hopping global optimization for the other sequences, alongside a structure from the Protein Databank (ID: 5OTU).^65^^b^ The lowest 200 minima located from each search were then used to seed the energy landscape explorations. The process is illustrated in Figure 1. GLP-1(7–36) structures were also studied, and the results can be found in Supporting Information. Structures were visualized with PyMOL.^66^

## Experimental Work

### GLP-1

GLP-1(7–37), H-HAEGTFTSDVSSYLEGQAAKEFIAWLVKGRG–OH, molecular weight 3355 Da, was purchased from GenScript in the form of an acetate salt with 98.1% purity. The residual content of the TFA salt was 0.94%, as determined by GenScript.

### Spectroscopy

Samples for circular dichroism (CD) and fluorescence measurements were freshly prepared at 100 μM GLP-1(7–37) in 25 mM citrate at pH 3 and 4 or 25 mM phosphate at pH 7.5. Circular dichroism spectra were measured on a Chirascan CD spectrometer (Applied Photophysics) in a 1 mm path length cuvette, and the measurement was performed with a 1 nm step size and with a 1 nm spectral bandwidth. The resulting spectrum was obtained as an average of three scans, and the spectrum of the pure buffer was subtracted. The CD machine units were converted to molar ellipticity using eq 1, where [θ]_molar_ is the molar ellipticity (with units deg cm^2^ dmol^–1^), *m*° is the CD signal in mdeg (machine units), *l* is the cuvette path length in cm, and *c* is the sample concentration in mol L^–1^.
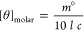
1

Intrinsic tryptophan fluorescence spectra were measured on a Cary Eclipse fluorescence spectrophotometer (Agilent Technologies). Spectra were obtained using an excitation wavelength of 280 nm, and emission spectra were recorded between 300 and 400 nm with a step of 1 nm. Emission and excitation band passes of 10 nm, and a voltage on the photomultiplier tube of 550 V were used. Samples were measured in a 120 μL quartz cuvette (Hellma Analytics). Fluorescence spectra were normalized. All measurements were performed at room temperature.

### Thioflavin T Binding Assays

The kinetics of peptide fibrillation was probed by thioflavin T (ThT) binding assays using a FLUOstar Omega microplate reader (BMG Labtech). GLP-1 samples of 25, 50, 75, and 100 μM in 25 mM citrate at pH 3 and 4 and in 25 mM phosphate at pH 7.5 were incubated with 50 μM ThT at 37 °C with agitation for 6 days. Peptide samples with ThT were incubated in a 96-well half area plate (Corning 3881) and sealed with tape (Costar Thermowell) to prevent samples from evaporating. The total volume of the sample in a well was 120 μL. The bottom reading of the plate was performed every 30 min with 5 min of shaking prior to each reading (orbital shaker mode at 600 rpm). ThT binding to fibrils and other species was monitored by recording the fluorescence emission at 482 nm after excitation at 448 nm. Fluorescence was measured at a gain of 500 with 8 flashes per well.

## Results and Discussion

### Potential and Free Energy Landscapes for Monomeric GLP-1

**Figure 2:**
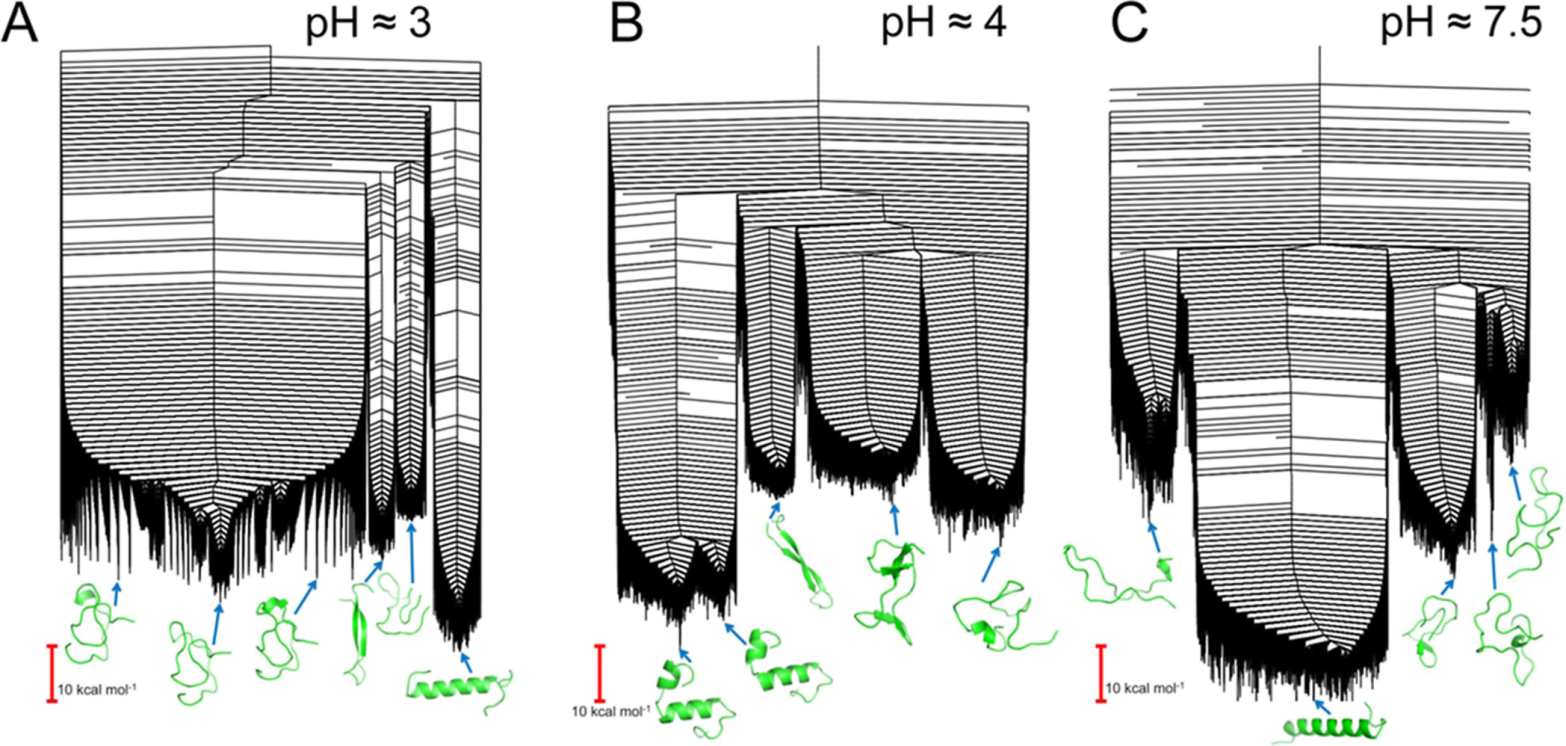
Disconnectivity graphs of the potential energy landscapes for monomeric GLP-1 at pH 3, 7–37^Prot3^ (A); pH 4, 7–37^Prot2^ (B); and pH 7.5, 7–37^Prot1^ (C). All landscapes are multifunneled, stabilizing multiple competing structural ensembles. Representative structures are provided for all major funnels.

The potential energy landscapes for the 7–37 variants of monomeric GLP-1 are shown in Figure 2, and those for the 7–36 variants in Figure S1. For the 7–37 variants, when the pH is 7.5, an α-helical structure^c^ is lowest in energy, and while other competing structures exist in other funnels, they are significantly higher in energy. When the pH is lowered to 4, instead of a single unbroken helix, we observe helical structures that are kinked, resulting in two shorter helical segments. Other structures are present in higher energy funnels, but again, there is a significant barrier in energy. At an even lower pH (around 3), the helical structure is the lowest again, and the kink has disappeared. The competing structural ensembles of more disordered structures, some with partial β-strands formed, are of comparable energy.

**Figure 3:**
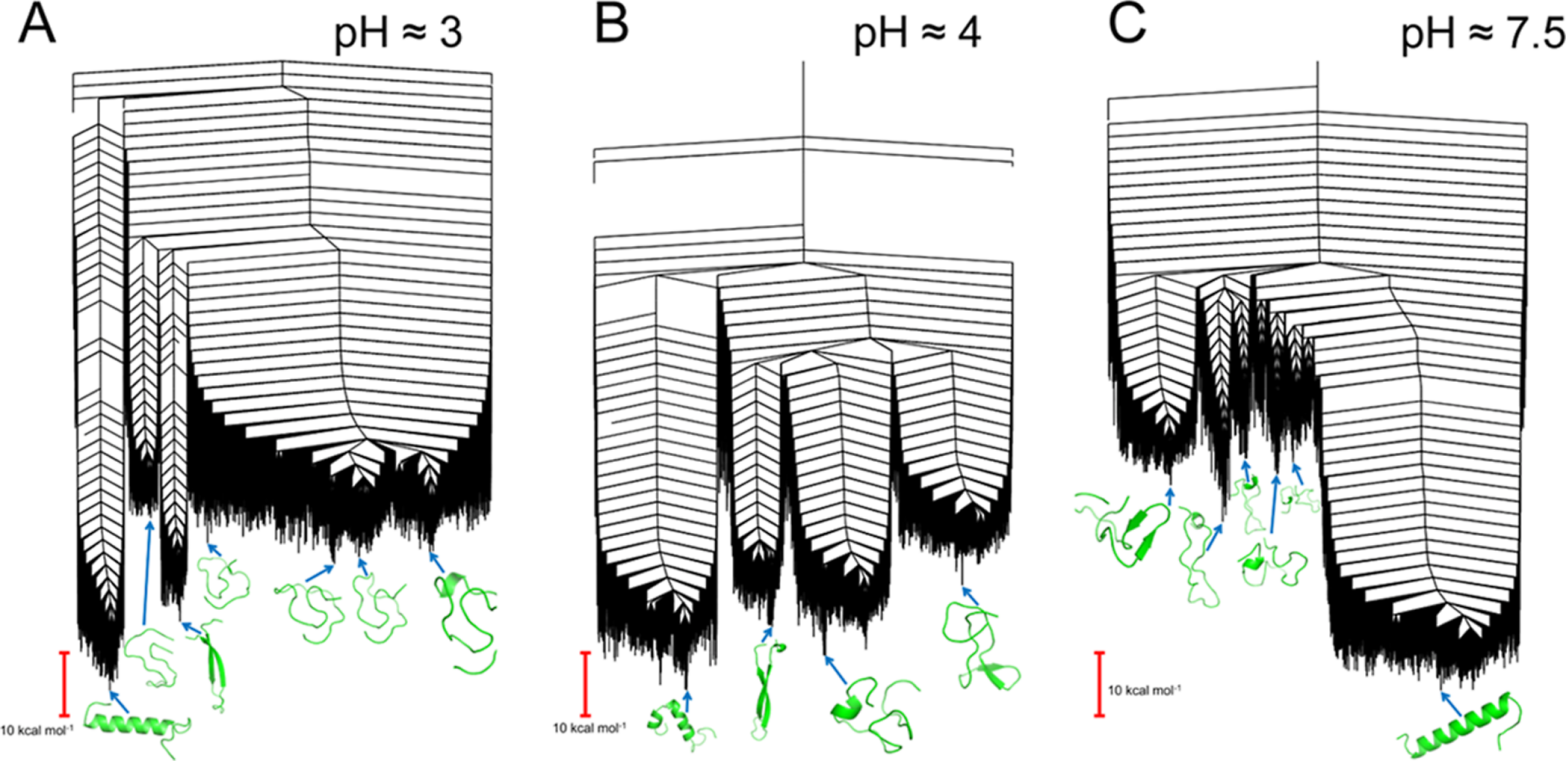
Disconnectivity graphs of the free energy landscapes for monomeric GLP-1 at pH 3, 7–37^Prot3^ (A); pH 4, 7–37^Prot2^ (B); and pH 7.5, 7–37^Prot1^ (C). All landscapes are multifunneled, stabilizing multiple competing structural ensembles. Representative structures are provided for all major funnels. Free energy landscapes constructed for *T* = 310 K.

The corresponding free energy disconnectivity graphs for the 7–37 variants at 310 K are shown in Figure 3, and the same data for the 7–36 variants are provided in Figure S2. With regard to the 7–37 variants, in all three conditions probed, the helical structures are lowest in free energy. However, there are significant pH-dependent changes in the relative energies of the competing structural ensembles and their accessibility. At near neutral pH (around 7.5), the funnel containing helical structures is significantly lower in free energy than the more disordered structural ensembles, and in all likelihood, this is the native monomeric fold. When the pH is decreased, the disordered structural ensembles become more accessible. While the helical structures are still the lowest in free energy, the lower free energy of the competing structures makes them more accessible. Apart from the kink observed in the helical structures at pH 4, the other key difference compared with pH 3 is the energy barriers between structural ensembles. In the more acidic conditions, the energy barriers between the helical structures and the disordered and β-strand structures are larger, resulting in more kinetic trapping and hence extended lifetimes.

### Characteristics of the Key Structural Ensembles

**Figure 4:**
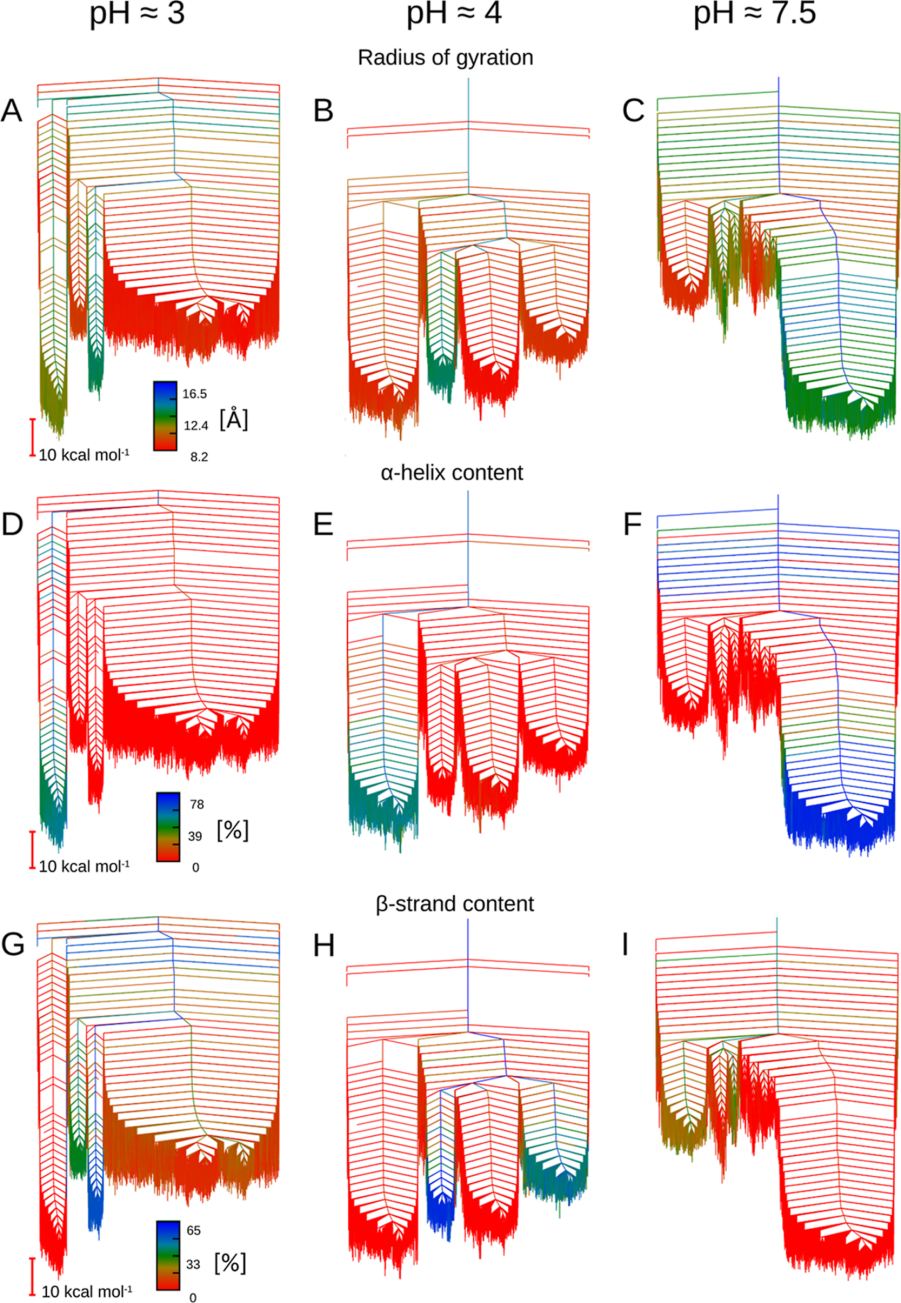
Disconnectivity graphs of the free energy landscapes for the 7–37 variants of monomeric GLP-1 at pH 3 (A,D,G), pH 4 (B,E,H), and pH 7.5 (C,F,I) colored using order parameters for key structural features. A, B, and C: radius of gyration is used as an order parameter, with compact structures in red and extended structures in blue and green. D, E, and F: order parameter is the α-helical content, where red is no helical content and green and blue are medium to high helical content. G, H, and I: β-strand content is used for coloring, with red indicating no β-strand content and green and blue indicating a medium and high level, respectively.

For an initial structural analysis, we used the radius of gyration and the α-helical and β-strand content to characterize each structure and then employed these values as a color scale for the free energy disconnectivity graphs (see Figure 4 for the 7–37 variants and Figure S3 for the 7–36 variants).

Regarding the 7–37 variants, for the radius of gyration, we observe an increase in compactness as the pH decreases. This observation is not surprising, given the extended nature of the helical structure at pH 7.5 alongside the significant energy difference between the disordered and helical structures at this pH. The analysis of the 7–36 variants, detailed in the Supporting Information, is more complicated.

Color-coding according to α-helical and β-strand content shows clearer trends, which are consistent between the 7–37 and 7–36 variants. For both variants, at high pH, the deepest and widest funnels consist of a multiplicity of structures with high α-helical content, and lowering the pH results in this funnel narrowing and the percentage α-helical content of individual structures becoming less pronounced. For the 7–37 variants, the α-helical content decreases from 68% at neutral pH to 52 and 48% at pH 3 and 4, respectively. Whereas β-strands are not observed at pH 7.5, two funnels arise at pH 4, one consisting of structures that are typically full β-strands and the other consisting of structures with partial β-strands. The latter structures typically disappear upon lowering the pH still further to 3, but the full β-strands are retained, functioning as the second-lowest energy structural type to the α-helix.

**Figure 5:**
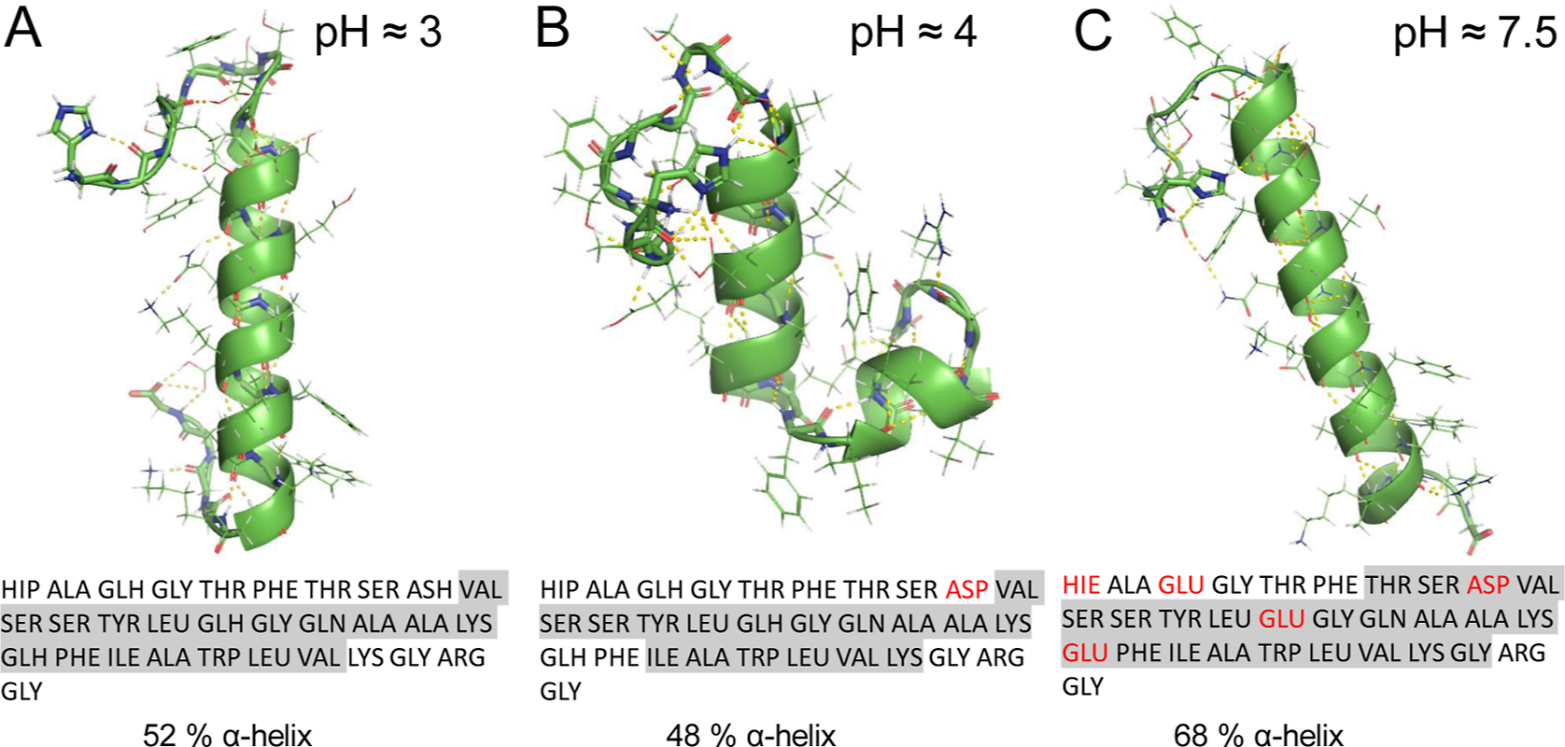
Lowest energy helical structures of monomeric GLP-1 (7–37 variant), at different protonation states. The sequence is provided for each structure. Residues in the α-helical region are highlighted in gray, and changes in the protonation states are highlighted in red.

To understand these changes in more detail, we looked at the changes in the protonation states of the individual residues at the different pH values and identified how these changes would impact intramolecular binding. The lowest energy helical structures from the energy landscapes for each pH condition for the 7–37 variants we probed are shown in Figure 5. The first point of interest here is the appearance of the kinked helical structures only at pH 4 but not at lower or higher pH values. The only protonation change from pH 3 to 4 occurs in the Asp9 side chain, which is deprotonated. The deprotonation allows for hydrogen-bonding of this side chain with the N-terminus, leading to changes in the orientations of the first nine residues. These changes in configuration lead to further interactions between the N-terminal tail and the C-terminal region of the helix. This set of interactions alters the side chain interactions in the helix and allows for interactions of the side chains of Tyr13 and Leu14 with other parts of the molecule. The interactions that are observed as a result are Tyr13-Arg30 and Leu14-Trp25. The former is more abundant, but we predict the coexistence of both structural ensembles due to their similar energies.

The protonation changes from pH 4 to 7.5 are more impactful, with deprotonation of Glu3, Glu15, and Glu21, introducing negative charges, and deprotonation of His1, which corresponds to the loss of its positive charge. As such, Glu15 is no longer capable of interacting with His1, which is a key interaction, holding the center of the chain to the N-terminus at pH 4. Furthermore, Glu21 can no longer interact with the carboxyl group at the C-terminus through the formation of a hydrogen-bond, again decreasing the likelihood of the α-helix forming a kink.

**Figure 6:**
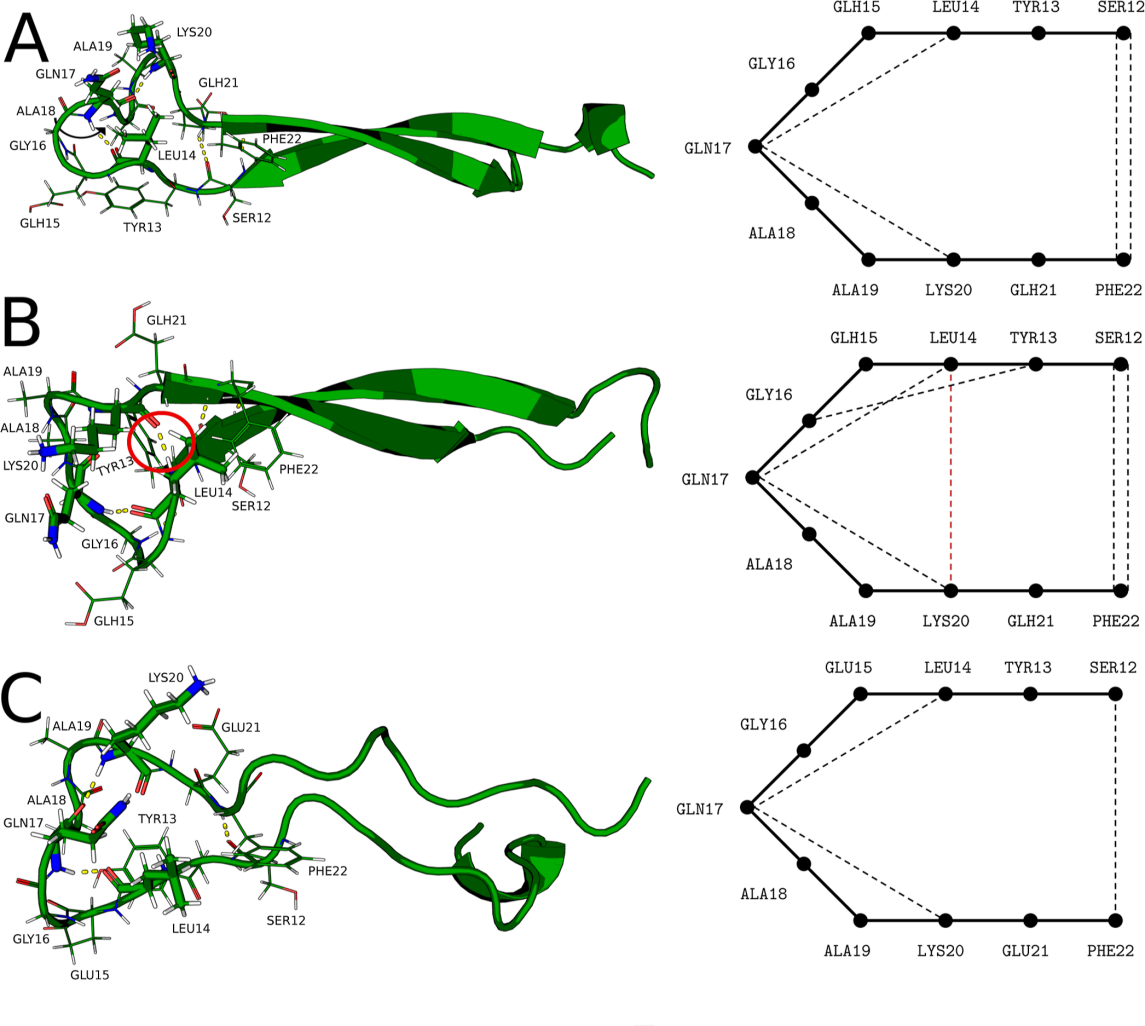
Lowest energy β-strand structures of monomeric GLP-1 (7–37 variant) at pH 3, 7–37^Prot3^ (A), pH 4, 7–37^Prot2^ (B), and pH 7.5, 7–37^Prot1^ (C). Residues Leu14, Gln17, and Lys20 are highlighted in licorice format, and all other β-turn residues are represented as sticks. All hydrogen-bonds between main-chain atoms are highlighted in yellow. Schematics of these hydrogen-bond networks are given on the right. The key Leu14–Lys20 interaction, which arises at pH 4, is highlighted in red both on the structure and in the schematic.

These changes in protonation states also explain the increased likelihood of β-strand formation at pH 4 vs neutral pH. The 7–37 variant at neutral pH exhibits the lowest energy β-strand structures with Glu21 (which is deprotonated) hydrogen-bonded to the amino group of Lys20. Lowering the pH to 4 causes protonation of Glu21, decreasing its capacity to interact with the Lys20 amino group. This loss causes the hydrophilic Lys20 to point further away from the cleft caused by the β-turn. This movement brings it into closer proximity to the functional group of Gln17, which in turn shifts away from the cleft in order to hydrogen-bond to the free amino group of Lys20. This movement further allows the β-hairpin to restructure, with the hydrophobic residue Leu14 occupying the space left in the cleft. In addition to this location being expressly stabilizing for Leu14, the main chain of this residue is thus brought into close proximity to the main chain of Lys20, allowing for the generation of a particularly stabilizing hydrogen-bond, as it establishes the β-hairpin as a more permanent, less dynamic motif. Further decreasing the pH to 3 shows that the Gln17–Lys20 hydrogen-bond is maintained and that Leu14 still occupies the β-hairpin cleft, but the main chains of Leu14 and Lys20 are no longer in close enough proximity to form a hydrogen-bond. Thus, the β-strand structure at pH 3 is stabilized by the occupation of the hairpin cleft by the hydrophobic Leu14 but not by the Leu14–Lys20 hydrogen-bond seen at pH 4, placing the stability of this structure between those of the β-strands at neutral pH and pH 4. This result is consistent with the trends shown by the disconnectivity graphs in Figure 4. These findings are summarized in Figure 6. Analysis of the 7–36 variant revealed an identical trend, as shown in Figure S4. The stabilization of the β-hairpin, instigated by the protonation of Glu21, thus explains why the β-strand motif becomes a stronger competitor to the main α-helix motif upon decreasing the pH to 4, with a marginal reversal of this effect when the pH decreases further beyond this value. Further discussion of the likely aggregation propensity of structures is provided in the Supporting Information (see Figure S6).

The schematics in Figures 6 and S4 follow the classification system for β-hairpins introduced by Milner-White & Poet.^67^ None of the GLP-1 variants studied can be neatly assigned to any of the sets proposed in this scheme, although for both 7–37^Prot3^ and 7–37^Prot2^ the class 1, seven-residue hairpin is the closest approximation. The fact that the neutral pH, 7–37^Prot1^ variant, cannot be assigned to a set at all is taken as further evidence that stable β-strand structures are not likely at this pH.

### Experimental Observations of pH-Dependence of GLP-1 Structures

**Figure 7:**
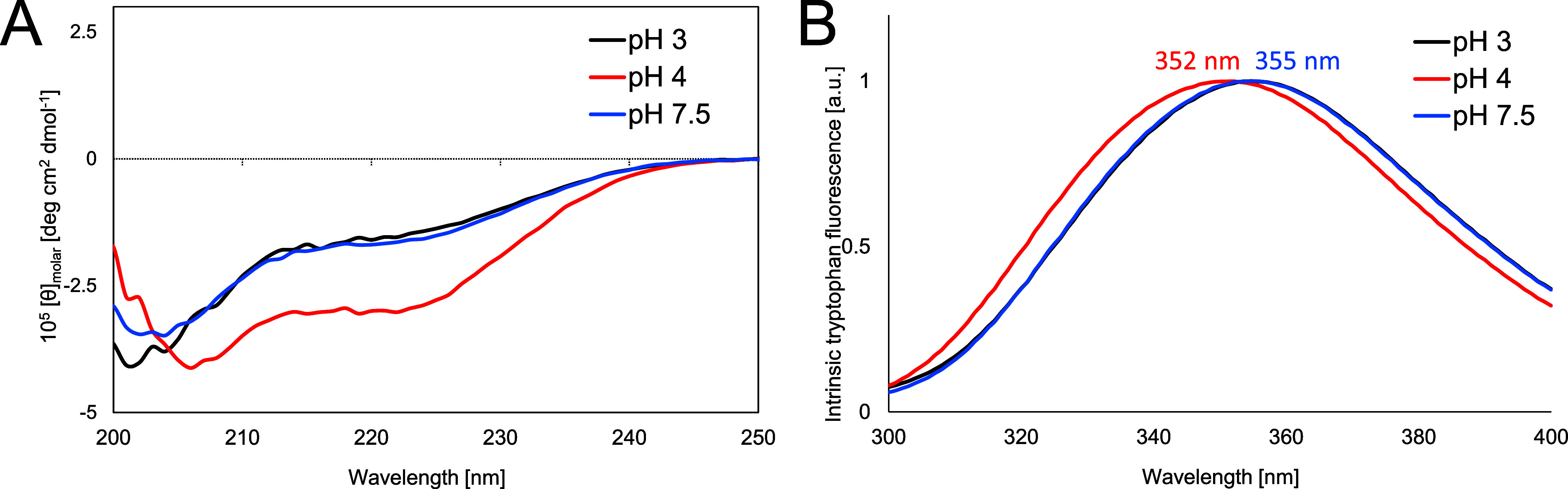
Far-UV CD and intrinsic tryptophan fluorescence of freshly prepared samples of GLP-1 (7–37) at pH 3, 4, and 7.5. Samples for CD (A) and fluorescence spectroscopy (B) were freshly prepared at 100 μM peptide concentration in 25 mM citrate at pH 3 and 4 or 25 mM phosphate at pH 7.5. Fluorescence spectra were recorded after an excitation at 280 nm.

The structural changes described above are also seen in experiments. Figure 7 shows the far-UV CD and fluorescence emission spectra of Trp25 for GLP-1 at the three different pH conditions. As is apparent from the CD signal intensity at 222 nm, the CD spectra exhibit a clear difference in the helical content for pH 4, whereas the spectra for pH 3 and 7.5 are very similar. This observation aligns well with the changes in the helical structures observed in the energy landscapes.

The fluorescence emission maximum for pH 4 is at 352 nm, which corresponds to a small blue-shift compared with the maxima for pH 3 and 7.5 at 355 nm. This result is also in agreement with the energy landscape picture. The fluorescence emission reflects the local environment of Trp25, and we infer from Figure 5B that the break in the helix for pH 4 leads to a more compact structure, allowing for additional interactions of Trp25. These interactions indicate that the residue is partially buried, leading to the observed shift.

### pH-Dependent Aggregation Propensities of GLP-1

**Figure 8:**
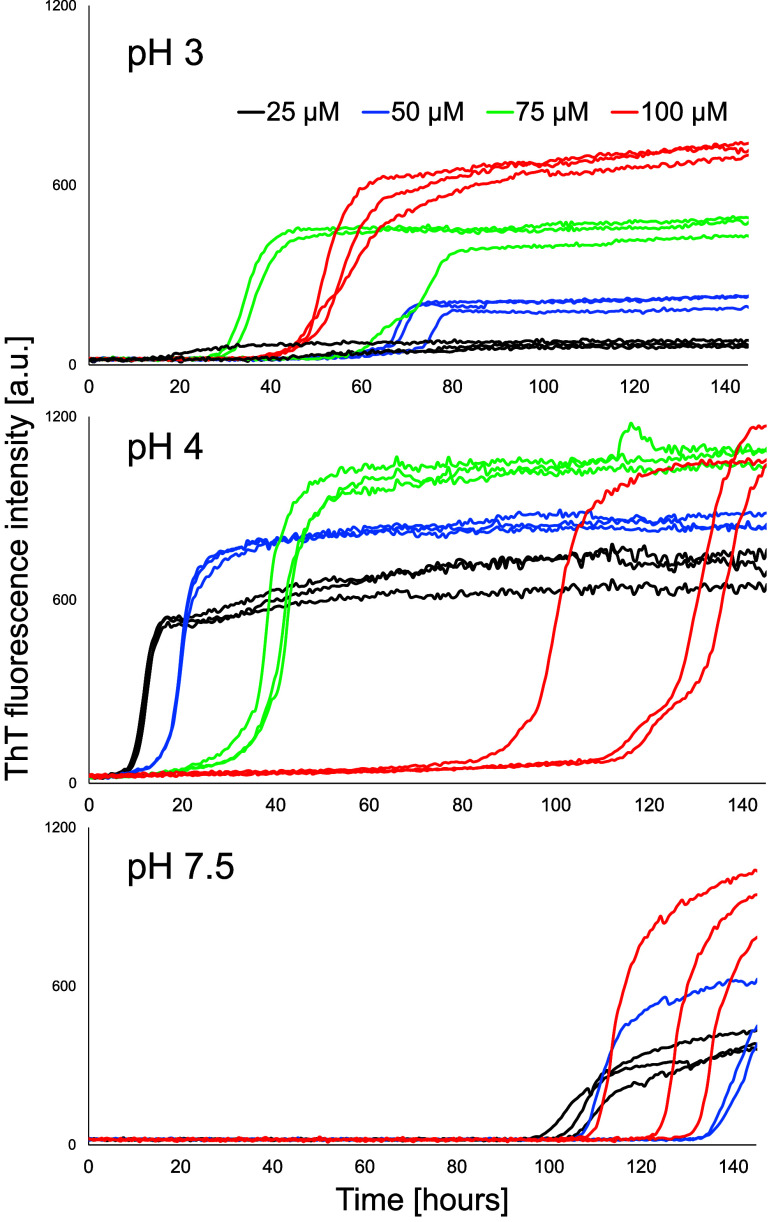
Thioflavin T assays of GLP-1 at pH 3 (top), 4 (middle), and 7.5 (bottom). GLP-1 samples were prepared in 25 mM citrate at pH 3 and 4 and in 25 mM phosphate at pH 7.5. Four different peptide concentrations were chosen for each pH: 25 μM (black), 50 μM (blue), 75 μM (green), and 100 μM (red). Our assay at pH 7.5 and concentration 75 μM failed, so we do not have measurements for this combination. Samples were incubated at 37 °C with agitation for 6 days. Fluorescence was recorded every 30 min at 482 nm after excitation at 448 nm.

Figure 8 shows GLP-1 aggregation kinetics as measured by ThT assay at three different pH conditions. The shortest lag time is observed for pH 4 at low GLP-1 concentrations (at higher GLP-1 concentrations, the aggregation is delayed due to the population of off-pathway species). At pH 3, aggregation is a little slower, but significant aggregation is still observed at higher GLP-1 concentrations within 2 days. In contrast, the lag time for the neutral pH sample is more than 4 days, which is a significant increase compared to lower pH values.

Earlier, we considered the free energy landscapes of the GLP-1 monomers. The structural ensemble properties observed in these landscapes match well with the aggregation propensities measured in the ThT assays. At pH 7.5, the free energy landscape features a significant stabilization of the helical structural ensemble compared to other clusters, including the β-strand-like structures. This energy difference means that the structural ensemble is biased toward helical structures, thus decreasing the concentration of the aggregation-competent β-forms. As a result, we observe longer lag times for the aggregation at neutral pH.

When the pH is lowered to 4, this energy difference is reduced, and β-strands are more likely formed. This change seems to stem both from the destabilization of the helical structures and the stabilization of the β-strands.

Finally, further lowering the pH to 3 stabilizes the helices again, and furthermore, we observe a large number of competing, non-β-strand structures. This structural composition seems to slow aggregation as observed via ThT assays compared to pH 4, but it is still significantly faster than for pH 7.5.

Interestingly, the aggregation behavior at pH 3 and 4 differs. At low GLP-1 concentrations, the peptide exhibits aggregation only at pH 4 in our assays. In contrast, at pH 3, aggregation at higher concentrations was faster than at lower concentrations and also faster than for pH 4. The maximum intensity of fluorescence was higher at pH 4, although a definitive conclusion that the amount of fibril formed is, therefore, greater at pH 4 cannot be drawn due to ThT fluorescence also varying with pH. The disconnectivity graphs in Figure 4 show that at pH 3, the overall barrier, which needs to be overcome for GLP-1 to change from minima in the α-helix funnel to minima in the β-strand funnel, is significantly higher than at pH 4. The fastest pathway for the interchange between the lowest-energy α-helix and the lowest energy β-strand was extracted from these respective data sets using Dijkstra’s algorithm,^68^ and the results (see Figure S5) show that the integrated path length at pH 4 is approximately one-quarter the value at pH 3. Together, this information suggests that, overall, the transformation from an α-helix structure to a β-strand structure is more likely at pH 4 than at pH 3, thus potentially explaining the higher maximum intensity of fluorescence at pH 4.

At pH 3, the funnel containing α-helical structures and the funnel containing complete β-strands are the lowest two. At pH 4, however, a large funnel consisting of disordered structures lies lower than that for complete β-strands. Hence, we infer that various different structural ensembles compete in this multifunnelled landscape, as is apparent from an examination of the disconnectivity graphs in Figures 3 and 4. Since the presence of off-pathway species appreciably reduces the rate of aggregation for GLP-1, and this effect is particularly pronounced at high peptide concentrations,^69^ these results may explain why the ThT assay shows the rate of aggregation decreasing with increasing peptide concentration at pH 4. At pH 3, where the generation of off-pathway competitors is not as prevalent, we would expect the ThT assay to show the opposite trend normally observed for the nucleation-propagation mechanistic, where the lag time decreases with increasing peptide concentration, which is indeed the case.

Care must be taken not to overinterpret the results from computational simulation. The force field and solvent model selected are standard for current peptide-based modeling, and, despite this combination being the recommendation of AMBER developers,^70^ a helical bias has been noted.^70,71^ To validate the choice of solvation model, the stability of distinct minima was tested with explicit solvent molecular dynamics simulations, which demonstated stability of the predicted structures (see Supporting Information, Figures S7 to S12). Restricting simulations to monomers only was a further limitation, as GLP-1 in solution coexists in an equilibrium of monomeric and oligomeric forms.^26^ However, for the purpose of this study, this restriction was necessary to prevent the energy landscapes from becoming prohibitively complicated. Nevertheless, the trends between experiment and simulation align very well. Given the difficulties associated with high-resolution structural analysis of peptides acting dynamically in solution, the energy landscape approach has therefore provided useful insights into the structural basis for the dependence of the aggregation propensity of GLP-1 on pH.

## Conclusions

We employed Energy Landscape Theory and associated computational tools to investigate and rationalize the conformational behavior of monomeric GLP-1 in different protonation states. All protonation states studied possess multifunnel energy landscapes with a variety of structurally different ensembles typical of intrinsically disordered and aggregation-prone proteins and peptides. α-Helical conformational ensembles had the lowest energy for all protonation states investigated. However, we find that at acidic pH values, β-structure-containing conformations are lower in energy, i.e., are more energetically favored relative to neutral pH. These observations are consistent with the experimental data, which, in general, exhibits a greater propensity for aggregation at acidic pH. Another trend, which was confirmed experimentally, is a structural change occurring in the α-helical ensemble between pH 3 and 4. This result provides validation that the computational methods capture important features of the conformational ensemble of GLP-1 at different pH values. We are therefore optimistic that this information will stimulate further study of GLP-1 aggregation, given the importance and potential of this peptide as a therapeutic. These results highlight the potential of the energy landscape approach to help rationalize experimental results, providing detailed structural information that is challenging to obtain experimentally, as well as predicting aggregation/misfolding propensities. We therefore suggest that this approach should be considered in the future study of pharmaceutical peptides.

## Supporting Information Available

Potential and free energy disconnectivity graphs for GLP-1(7–36) at different pH and analysis of radius of gyration and secondary structure content, β-strand classification for GLP-1(7–36), Dijkstra’s shortest path analysis for GLP-1(7–37), and explicit solvent MD simulations for selected structures (PDF)

## Footnotes

a New configurations for each basin-hopping step are created by altering the configuration of a selected rotamer.

b The pdb structure is for GLP-1(7–36), and we added the missing residue.

c All subsequent references to helices in this paper refer exclusively to α-helices.
