# Supplementary material for: Energy Landscapes and Structural Ensembles of Glucagon-like Peptide-1 Monomers
Source: J Phys Chem B. 2024 Jun 4;128(23):5601–11. doi: 10.1021/acs.jpcb.4c01794 (PMC11182347; doi:10.1021/acs.jpcb.4c01794)
Supplement: Supplementary file 1 — jp4c01794_si_001.pdf [file jp4c01794_si_001.pdf]

Energy Landscapes and Structural Ensembles of Glucagon-Like Peptide-1 Monomers

Alasdair D. Keith,[†,‡,∥] Eva Přáda Brichtová,[†,¶,∥] Jack G. Barber,[†] David J. Wales,[†] Sophie E. Jackson,[*,†] and Konstantin Röder[*,†,§]

†*Yusuf Hamied Department of Chemistry, University of Cambridge, Cambridge, United Kingdom*

‡*Now: Department of Biochemistry, School of Medicine, Emory University, Atlanta, GA, USA*

¶*Now: Institute of Chemical, Environmental and Bioscience Engineering, Technische Universität Wien, Vienna, Austria*

§*Now: Randall Centre for Cell & Molecular Biophysics, King's College London, London, United Kingdom*

∥*Contributed equally to this work*

E-mail: sej13@cam.ac.uk; konstantin.roeder@kcl.ac.uk

Starting Points and Force Field for Simulations for GLP-1 (7-36)

The starting points for GLP-1 (7-36) simulations were generated using the same methods used for GLP-1 (7-37) described in the main paper. Protein Databank structure 5OTU[1] was used as a seed for basin-hopping global optimization. The protonation states studied are provided in Table S1.

Table S1: Sequence and protonation states of GLP-1 (7-36) variants. Approximate pH values are given so that simulations can be more readily compared to experiment. Three-letter labels for residues indicating the state of protonation/deprotonation follow the convention developed by the AMBER package. Here, histidine is represented not by HIS, but by HID if the residue is deprotonated and has a hydrogen on the delta nitrogen only, by HIE if the residue is deprotonated and has a hydrogen on the epsilon nitrogen only, and by HIP is the residue is protonated and so has hydrogen atoms on both the delta and epsilon nitrogens. GLU represents glutamic acid in its deprotonated form whereas GLH represents this residue when protonated. Residues highlighted in red are those which differ from 7-36$^{\mathrm{Prot1}}$.

Label	Approximate pH	Sequence
7-36$^{\mathrm{Prot1}}$	7.5	`HIE ALA GLU GLY THR PHE THR SER ASP VAL SER SER TYR LEU GLU GLY GLN ALA ALA LYS GLU PHE ILE ALA TRP LEU VAL LYS GLY ARG`
7-36$^{\mathrm{Prot2}}$	7.5	`HID ALA GLU GLY THR PHE THR SER ASP VAL SER SER TYR LEU GLU GLY GLN ALA ALA LYS GLU PHE ILE ALA TRP LEU VAL LYS GLY ARG`
7-36$^{\mathrm{Prot3}}$	$4 \leq x < 7.5$	`HIP ALA GLU GLY THR PHE THR SER ASP VAL SER SER TYR LEU GLU GLY GLN ALA ALA LYS GLU PHE ILE ALA TRP LEU VAL LYS GLY ARG`
7-36$^{\mathrm{Prot4}}$	4	`HIP ALA GLH GLY THR PHE THR SER ASP VAL SER SER TYR LEU GLH GLY GLN ALA ALA LYS GLH PHE ILE ALA TRP LEU VAL LYS GLY ARG`

GLP-1 (7-36) PE Disconnectivity Graphs

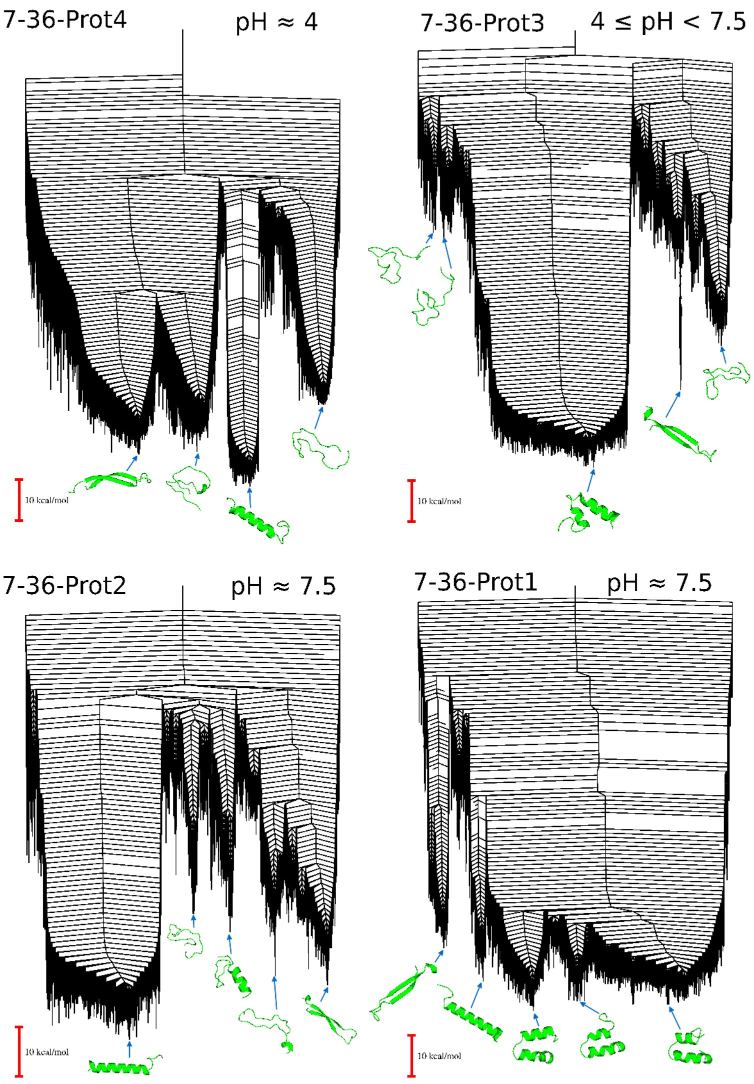

Figure S1: Disconnectivity graphs of the potential energy landscapes for monomeric 7-36 GLP-1 in selected protonation states. Top left: 7-36$^{\mathrm{Prot4}}$, pH 4. Top right: 7-36$^{\mathrm{Prot3}}$, 4 $\leq$ pH < 7.5. Bottom left: 7-36$^{\mathrm{Prot2}}$, pH 7.5. Bottom right: 7-36$^{\mathrm{Prot1}}$, pH 7.5. All landscapes are multifunneled, stabilising multiple competing structural ensembles. Representative structures are provided for all major funnels.

GLP-1 (7-36) FE Disconnectivity Graphs

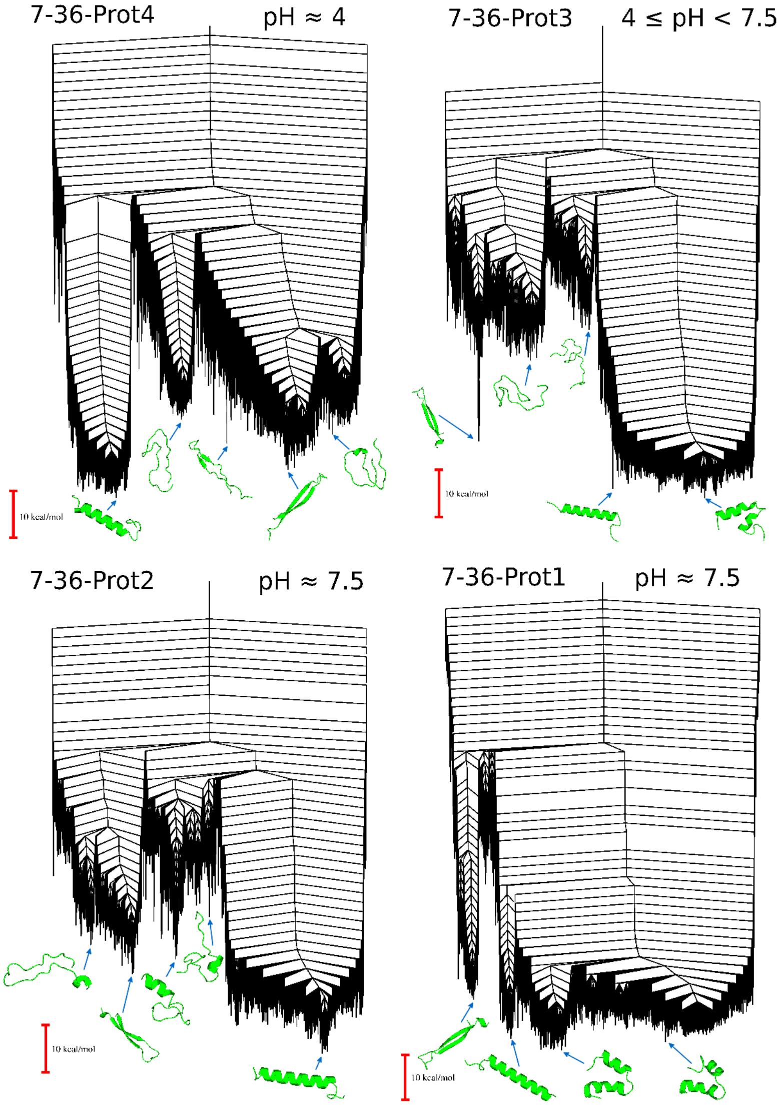

Figure S2: Disconnectivity graphs of the free energy landscapes for monomeric 7-36 GLP-1 in selected protonation states. Top left: 7-36$^{\mathrm{Prot4}}$, pH 4. Top right: 7-36$^{\mathrm{Prot3}}$, $4 \leq \mathrm{pH} < 7.5$. Bottom left: 7-36$^{\mathrm{Prot2}}$, pH 7.5. Bottom right: 7-36$^{\mathrm{Prot1}}$, pH 7.5. All landscapes are multi-funneled, stabilising multiple competing structural ensembles. Representative structures are provided for all major funnels.

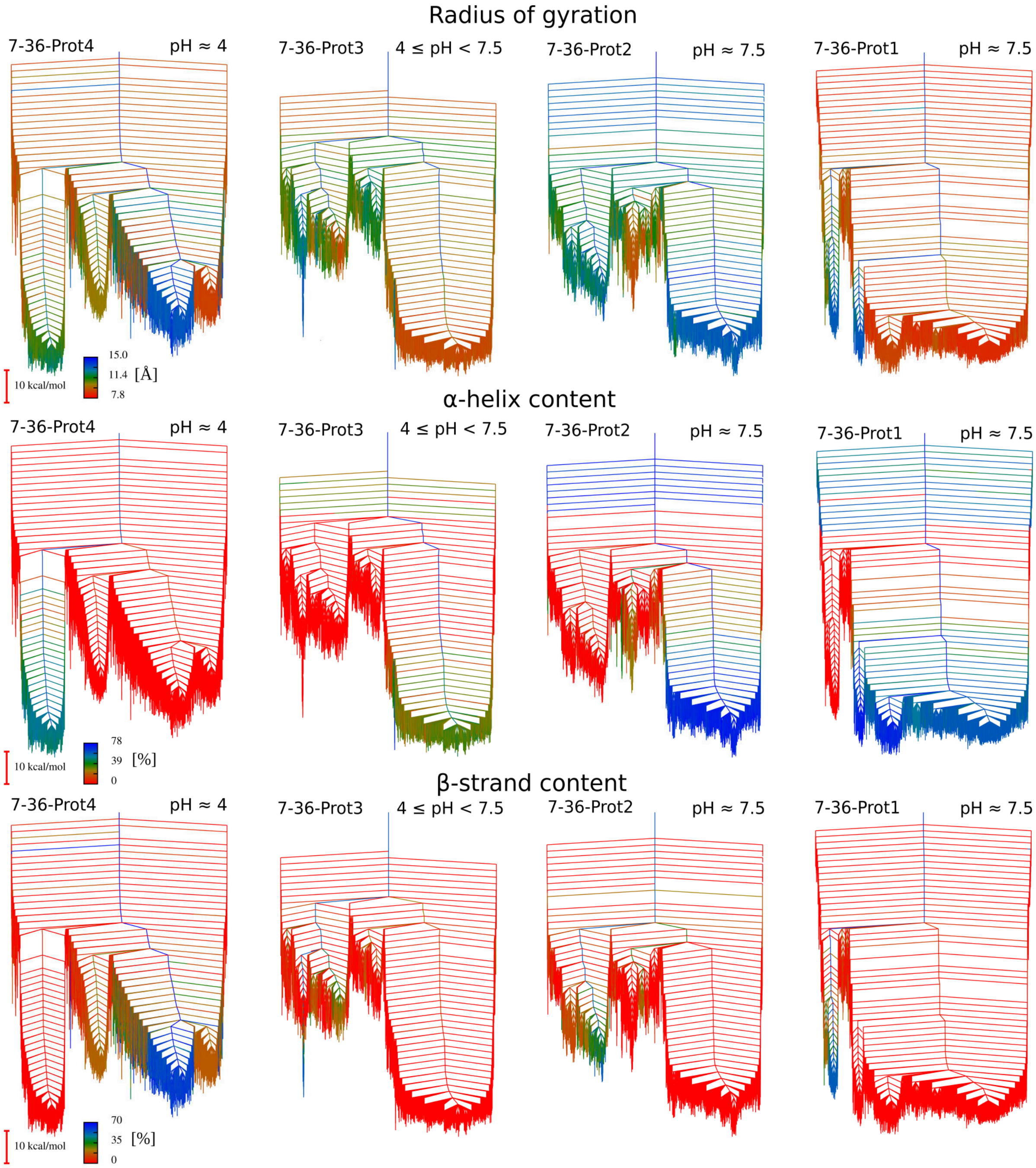

Figure S3: Disconnectivity graphs of the free energy landscapes for monomeric 7-36 GLP-1, coloured using order parameters for key structural features. Top row: The radius of gyration is used as the order parameter, with compact structures in red and extended structures in blue and green. Middle row: The order parameter is the α-helical content, where red is no helical content and green and blue are medium to high helical content. Bottom row: The β-strand content is used for colouring, with red indicating no β-strand content, and green and blue indicating a medium and high level, respectively.

GLP-1 (7-36) β-strand Classification

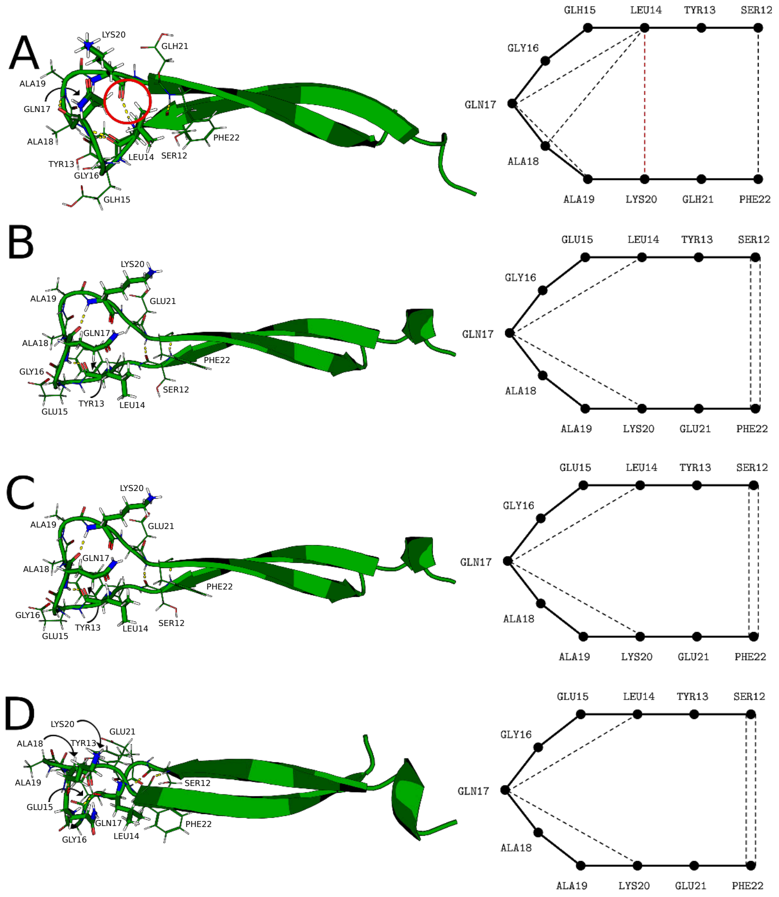

Figure S4: Lowest energy β-strand structures of monomeric GLP-1 (7-36 variant) at pH 4, 7-36$^{\text{Prot4}}$ (A), $4 \leq$ pH < 7.5, 7-36$^{\text{Prot3}}$ (B), pH 7.5, 7-36$^{\text{Prot2}}$ (C) and pH 7.5, 7-36$^{\text{Prot1}}$ (D). Residues Leu14, Gln17 and Lys20 are highlighted in licorice format, and all other β-turn residues are represented as sticks. All hydrogen bonds between main-chain atoms are highlighted in yellow. Schematics of these hydrogen bond networks are given on the right. The key Leu14-Lys20 interaction which arises at pH 4 is highlighted in red on both the structure and in the schematic.

GLP-1 (7-36) Discussion

Radius of Gyration (*see row 1 of Fig. S3*): At pH 7.5, the compactness strongly depends upon which of the δ and ϵ nitrogen atoms of residue 1 (a histidine) are protonated. Lowering the pH so that both of these nitrogen atoms become protonated results in a generally compact structure. However, upon lowering the pH still further, so that the glutamic acid residues also become protonated, results in a predominance of extended α-helix and β-strand structures.

α-helical *vs* β-strand Content (*see rows 2 and 3 of Fig. S3*): At high pH, the deepest and widest funnel consists of a multiplicity of structures with high α-helical content, and lowering the pH results in this funnel narrowing and the percentage α-helical content of individual structures becoming less pronounced. β-strands are observed at neutral pH, but they only consist of a small proportion of structures. Upon lowering the pH to 4, these β-strands become more prevalent, and this structure type forms the second-lowest energy funnel (like the 7-37 case), with the α-helix type forming the lowest.

GLP-1 (7-37) Dijkstra Fastest Pathways

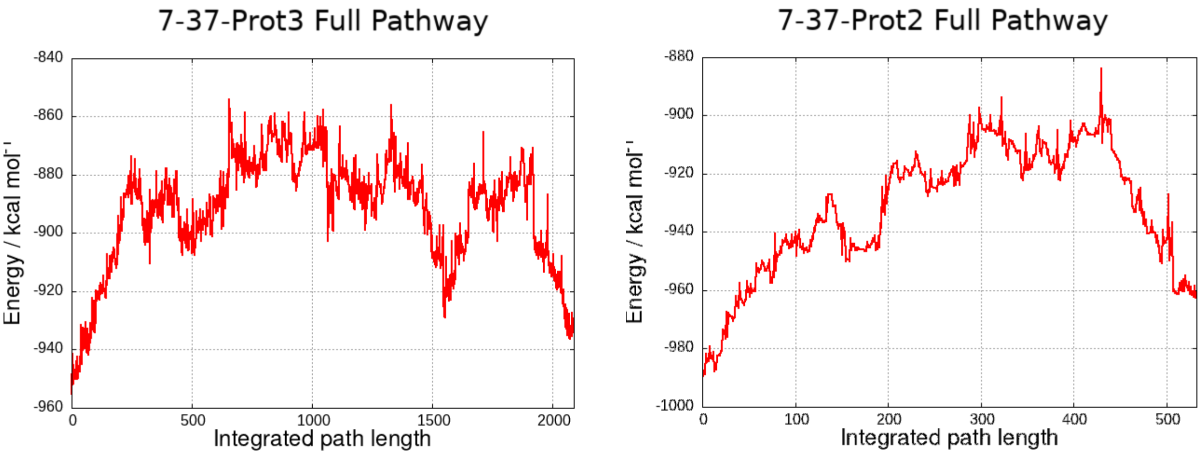

Figure S5: Dijkstra fastest pathways between the lowest energy α-helix and β-strand structures for the 7-37 GLP-1 variant at pH 3 (left) and pH 4 (right). At pH 4, the integrated path length is only 531 stationary points, versus 2087 at pH 3.

Analysis of aggregation-prone configurations

Amyloidal aggregation generally proceeds via the adoption of assembly-competent monomeric states, so called N* states. Analysis of the structural ensembles to identify these states and their propensities can reveal insight into the likelihood of aggregation, as demonstrated for amyloid-β in previous studies.[2,3] Such an analysis requires knowledge of the amyloidal core region, so that a comparison can be made between the eventual fold in the fibril and the monomeric structures.

To our knowledge, such a structure is not publicly available for GLP-1, although some structures of aggregating fragments are available. This limitation means we could not conduct a full N* analysis, but instead compared the structural similarity across the landscapes to one of the reported peptide structures. The peptide structure used for comparison is PDB 8ONQ,[4] a seven residue peptide exhibiting an anti-parallel β-sheet-like structure.

Figure S6 shows the energy landscapes for GLP-1 (7-37) using the backbone RMSD to

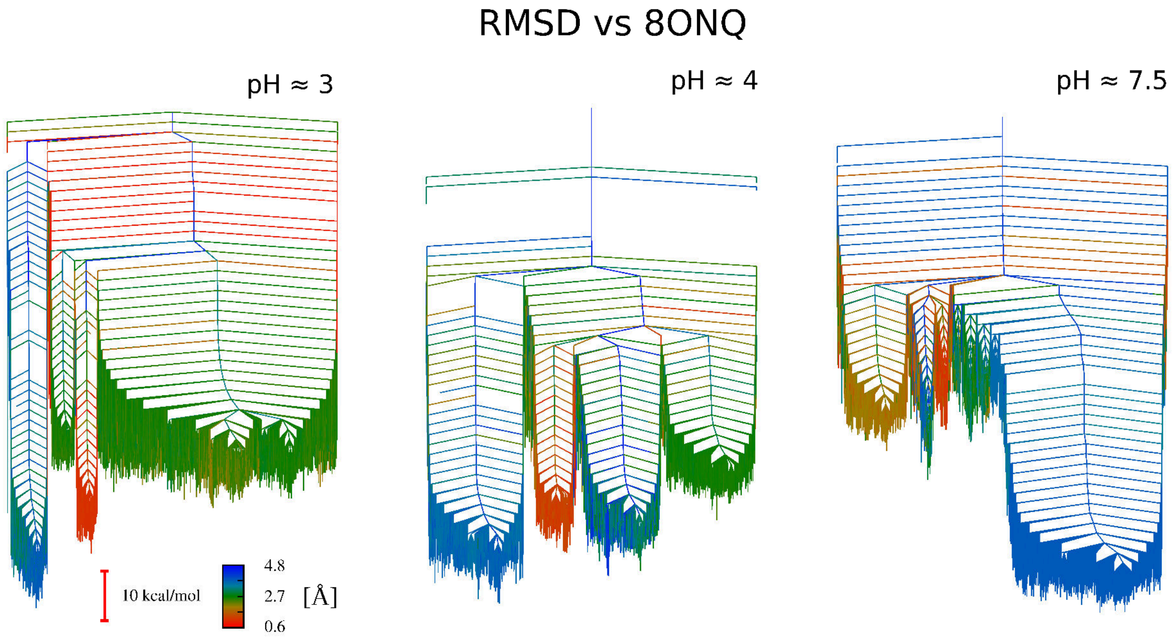

Figure S6: The energy landscapes for 7-37 GLP-1 at the three pH values coloured by the backbone RMSD to 8ONQ. The colouring closely resembles the β-strand content and indicates the higher stability of aggregation-prone species at lower pH.

8ONQ as the colouring scheme. The resulting disconnectivity graphs resemble the ones for the β-strand content closely. This finding is not surprising, given the strong β-strand character of the aggregated peptide. A higher availability of aggregation-prone structures at lower pH is observed.

Explicit solvent simulations

For each landscape for GLP-1 (7-37), we selected four minima representative of the different funnels for explicit solvent molecular dynamics (MD) simulations to validate their stability. Each structure was solvated in OPC water in a truncated octahedral solvation box with a distance of 8.0 Å to the box surface from the solute. Cl^- ions were added to give an effective concentration of 0.15 M, and K^+ ions were used to neutralise the system.

The energy of the system was first minimised with and without constraints on the solute, before the simulation box was heated to 300 K with restraints on the solute. These restraints

were removed stepwise, before the simulation box was equilibrated in an NPT ensemble. We then ran three 50 ns production trajectories for each minimum.

For each pH value, one minimum is helical (185102 for pH 7.5, 23216 for pH 4 and 131790 for pH 3), while the other three are disordered with varying secondary structure content.

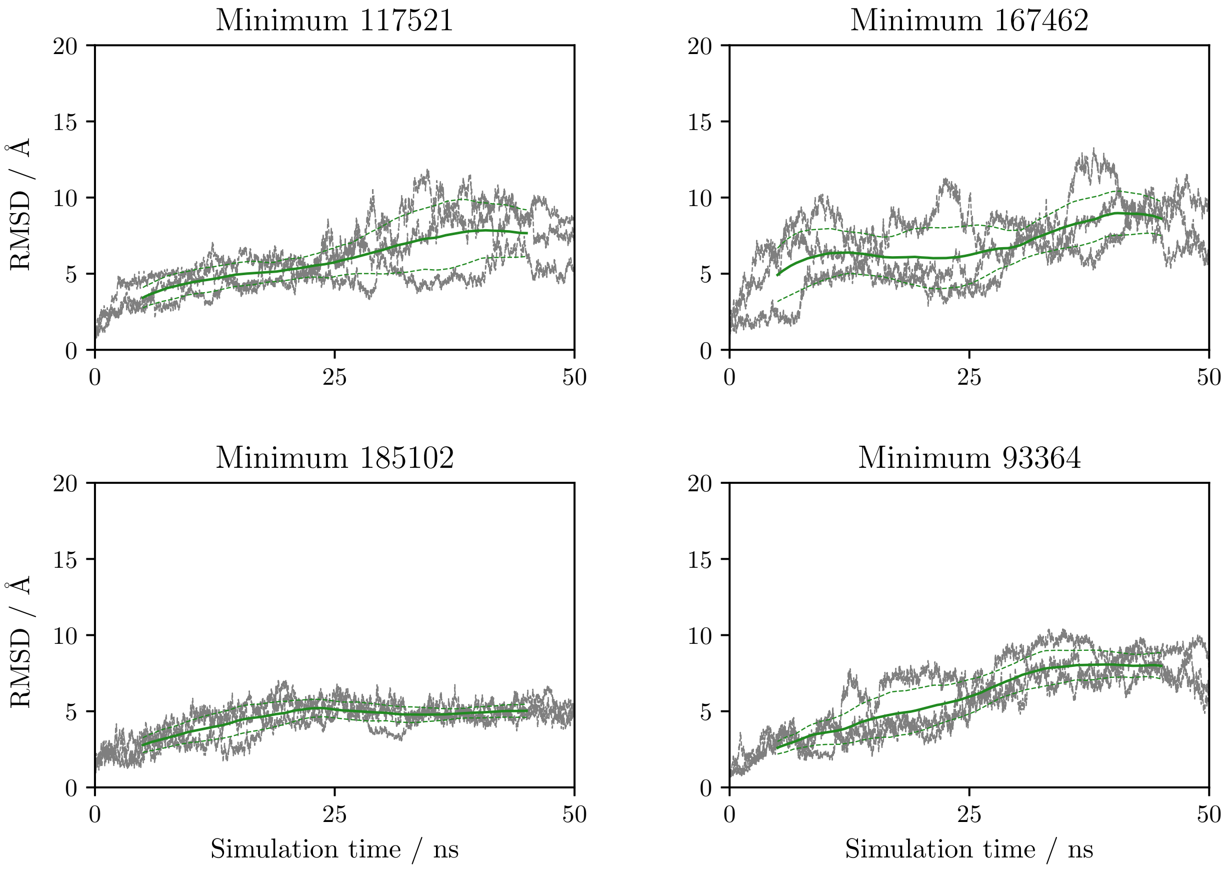

Figure S7: Backbone RMSD changes during explicit solvent MD simulations for four minima at pH 7.5. The individual trajectories are shown in grey, with the average and standard deviation in green. No larger structural changes are observed on the MD time scale.

Figures S7 to S9 show the changes in backbone RMSD during the simulations. We observe no large structural fluctuations. This observation is further supported by an analysis of the secondary structure content across the trajectories using DSSP.[5] Figures S10 to S12 give the average secondary structure for each residue. Helical and β-strand like structures are preserved in the MD simulations. Furthermore, the disordered structures do not adopt alternative folded structures.

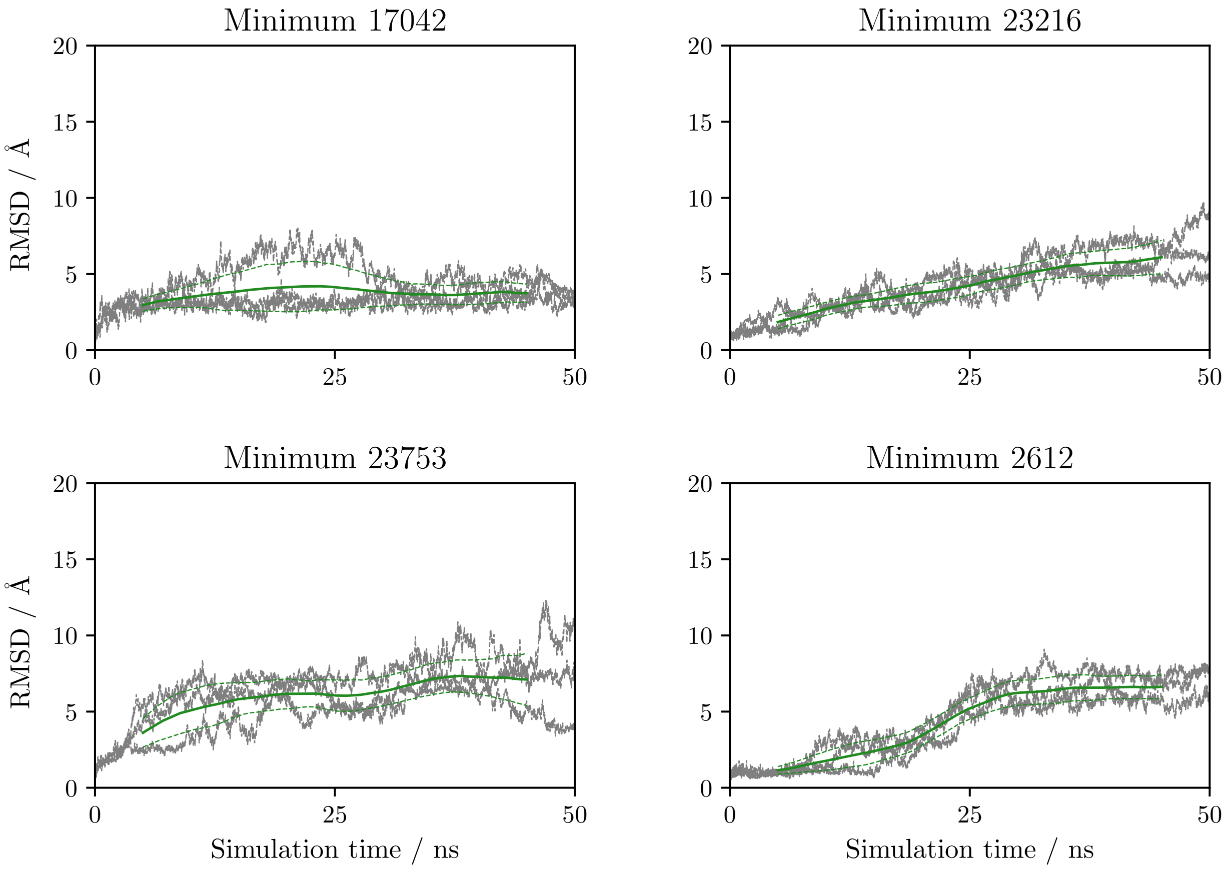

Figure S8: Backbone RMSD changes during explicit solvent MD simulations for four minima at pH 4. The individual trajectories are shown in grey, with the average and standard deviation in green. No larger structural changes are observed on the MD time scale.

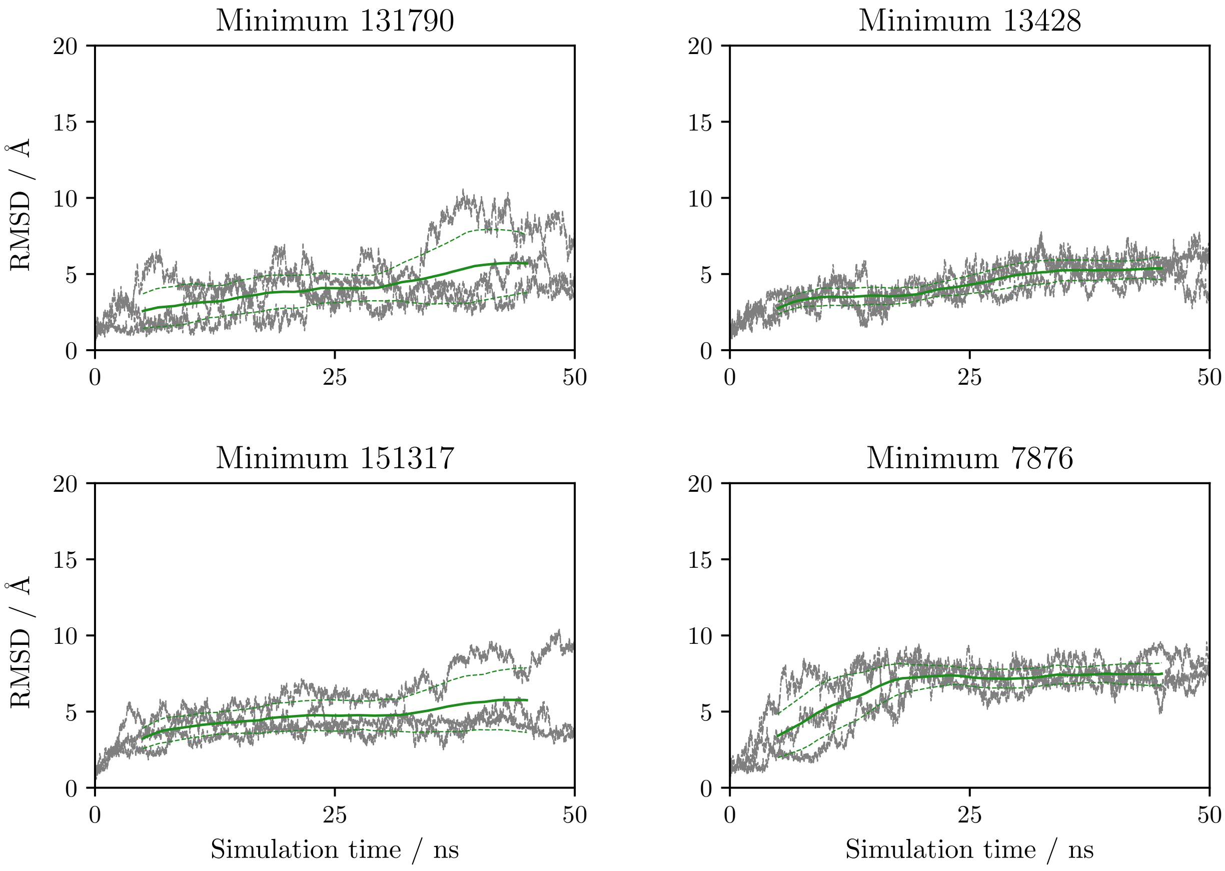

Figure S9: Backbone RMSD changes during explicit solvent MD simulations for four minima at pH 3. The individual trajectories are shown in grey, with the average and standard deviation in green. No larger structural changes are observed on the MD time scale.

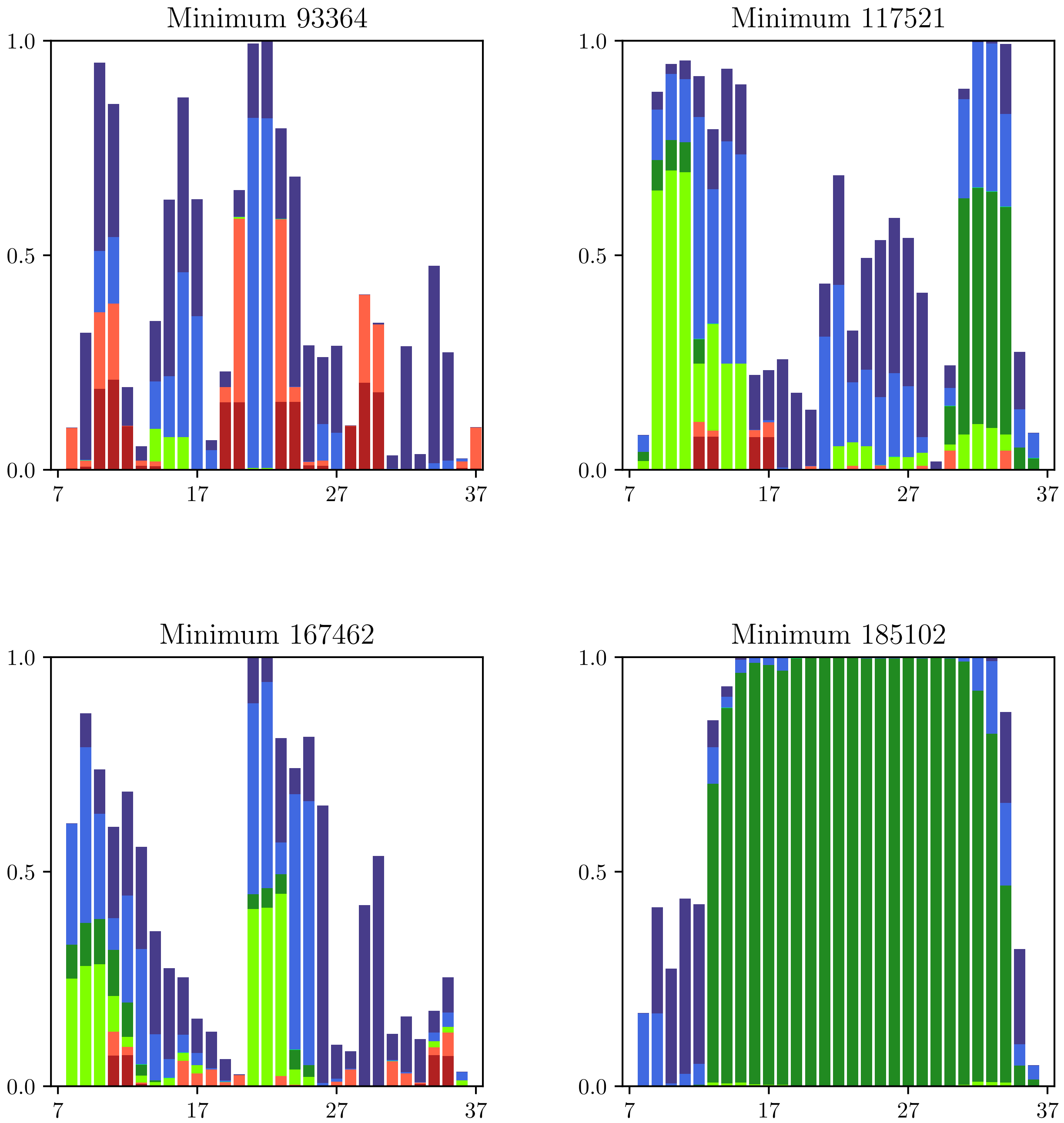

Figure S10: Secondary structure averaged over the entire set of trajectories for each minimum at pH 7 for each residue. Dark and light red are parallel and antiparallel β-strand configurations, dark green an α-helix, light green a 3-10 helix, and dark and light blue are turns and bends, respectively.

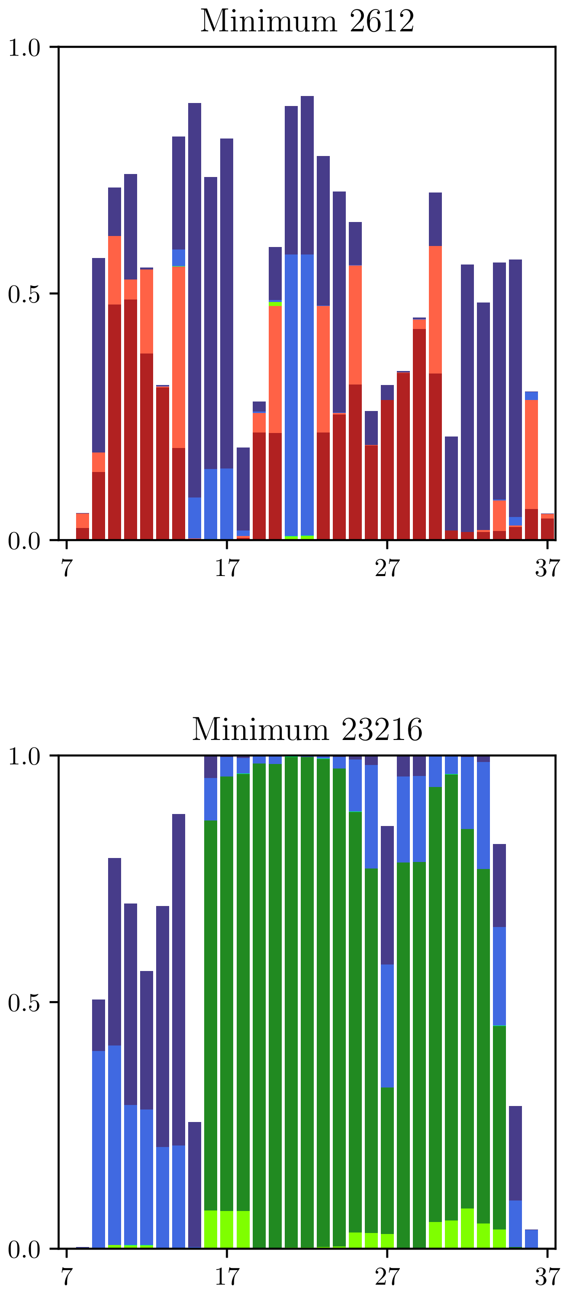

Figure S11: Secondary structure averaged over the entire set of trajectories for each minimum at pH 4 for each residue. Dark and light red are parallel and antiparallel β-strand configurations, dark green an α-helix, light green a 3-10 helix, and dark and light blue are turns and bends, respectively.

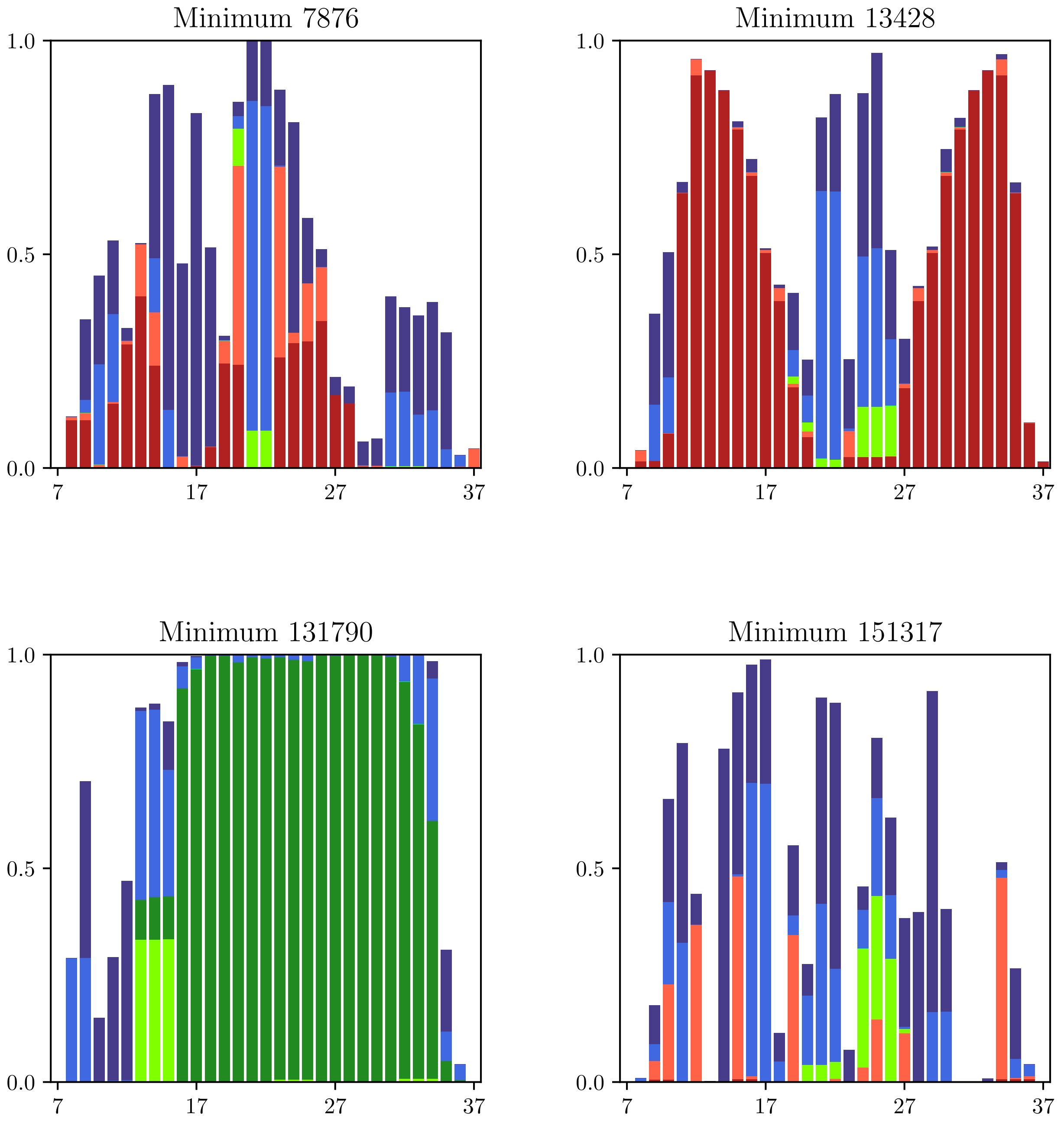

Figure S12: Secondary structure averaged over the entire set of trajectories for each minimum at pH 3 for each residue. Dark and light red are parallel and antiparallel β-strand configurations, dark green an α-helix, light green a 3-10 helix, and dark and light blue are turns and bends, respectively.

References

(1) Oddo, A.; Mortensen, S.; Thøgersen, H.; De Maria, L.; Hennen, S.; McGuire, J. N.; Kofoed, J.; Linderoth, L.; Reedtz-Runge, S. α-Helix or β-Turn? An Investigation into N-Terminally Constrained Analogues of Glucagon-like Peptide 1 (GLP-1) and Exendin-4. *Biochem.* **2018**, *57*, 4148–4154.

(2) Tarus, B.; Straub, J. E.; Thirumalai, D. Dynamics of Asp23Lys28 Salt-Bridge Formation in A10-35 Monomers. *J. Am. Chem. Soc.* **2006**, *128*, 1615916168.

(3) Chakraborty, D.; Straub, J. E.; Thirumalai, D. Energy landscapes of Aβ monomers are sculpted in accordance with Ostwalds rule of stages. *Sci. Adv.* **2023**, *9*, eadd6921.

(4) Polymorphic amyloid nanostructures of hormone peptides involved in glucose homeostasis display reversible amyloid formation. *Nat. Commun.* **2023**, *14*, 4621.

(5) Kabsch, W.; Sander, C. Dictionary of protein secondary structure: Pattern recognition of hydrogen-bonded and geometrical features. *Biopolymers* **1983**, *22*, 2577–2637.
